# Effect of Transcranial Direct Current Stimulation Combined With Xbox-Kinect Game Experience on Upper Limb Movement in Down Syndrome: A Case Report

**DOI:** 10.3389/fbioe.2020.00514

**Published:** 2020-05-29

**Authors:** Jamile Benite Palma Lopes, Isabela Marques Miziara, Manuela Galli, Veronica Cimolin, Claudia Santos Oliveira

**Affiliations:** ^1^Health Sciences Program, Faculty of Medical Sciences of Santa Casa de São Paulo, São Paulo, Brazil; ^2^Undergraduate Department, Faculty of Taquaritinga - FTGA, Taquaritinga, Brazil; ^3^Undergraduate Department, Faculty of Electrical Engineering, Federal University of Uberlândia, Uberlândia, Brazil; ^4^Undergraduate Department, Faculty of Electrical and Biomedical Engineering, Federal University of Pará, Belém, Brazil; ^5^Department of Electronics, Information and Bioengineering, Politecnico di Milano, Milan, Italy; ^6^Program in Human Movement and Rehabilitation Center of Anápolis, Anápolis, Brazil; ^7^Master's and Doctoral Program in Health Sciences, São Paulo Santa Casa School of Medical Sciences, São Paulo, Brazil

**Keywords:** down syndrome, physical therapy, upper limb, reaching, electrical stimulation, motor cortex

## Abstract

Transcranial direct current stimulation (tDCS) is a non-invasive brain stimulation technique used to enhance local synaptic efficacy and modulate the electrical activity of the cortex in neurological disorders. Researchers have sought to combine this type of stimulation with well-established therapeutic modalities, such as motor training involving Xbox Kinect games, which has demonstrated promising results. Thus, this study aimed to determine whether tDCS can enhance upper limb motor training in an eight-year-old child with Down Syndrome (DS) (cognitive age: five years, based on the Wechsler Intelligence Scale for Children). The evaluations consisted of three-dimensional analysis of upper limb kinematics during a reaching task performed before, after10 session, and one month after the intervention. The intervention protocol involved 1 20-min sessions of tDCS over the primary motor cortex at an intensity of 1 mA during Xbox Kinect game training involving an upper limb motor task. The analysis of the kinematic data revealed that in the pre-intervention evaluation, the dominant limb executed the task slowly and over a long path. These aspects improved at the post-intervention and follow-up evaluations, as demonstrated by the shorter total movement duration (3.05 vs. 1.58 vs. 1.52 s, respectively). Similar changes occurred with the non-dominant upper limb; a significant increase in movement velocity at the post-intervention and follow-up evaluations was observed (0.53 vs. 0.54 vs. 0.85 m/s, respectively). The present case report offers preliminary data from a protocol study, and the results confirm the notion that anodal tDCS combined with upper limb motor training leads to improvements in different kinematic variables.

## Introduction

Human beings have 46 chromosomes that separate during the process of cell division. However, this division does not always take place correctly, and some chromosome pairs do not divide. This event in chromosome 21 is known as trisomy 21 and is characterized by the “non-disjunction” or “non-separation” of the chromosome; this is the most common form of Down syndrome (DS) (Dennis, 1995; Moreira et al., 2000). Two other chromosome anomalies are related to DS, namely, translocation and mosaicism. Translocation is more frequent and is characterized by an extra chromosome of pair 21 that is united with a chromosome from another pair. Although individuals with this anomaly have 46 chromosomes, they have DS. The less frequent and more severe form is mosaicism, which occurs due to a genetic abnormality that compromises only some of the cells; that is, some cells have 46 chromosomes, and some have 47 (Korenberg et al., 1990, 1994; Epstein et al., 1991; Korenberg, 1991; Dennis, 1995; Patterson, 1995; Chapman and Hesketh, 2000; Moreira et al., 2000)

Chromosome anomalies lead to an increase in the protein expression of genes, which exerts considerable effects on brain development (Korenberg et al., 1990, 1994; Epstein et al., 1991; Korenberg, 1991; Dennis, 1995; Patterson, 1995; Chapman and Hesketh, 2000; Moreira et al., 2000). According to the literature, the population with DS has both structural and functional abnormalities of the nervous system, such as changes in the shape and number of neurons, a smaller brain volume, and maturation disorders, as well as physiopathological processes, such as a reduction in release of neurotransmitters and degenerative processes of the nervous system (Malak et al., 2015; Steve et al., 2015). The smaller brain volume in the population with DS can lead to significant psychomotor impairment, affecting cognition, voluntary movement, and gait quality (Pinter et al., 2001; Stefan et al., 2004).

Encephalic hypoplasia, especially in the cerebellum, is common in this population and leads to muscle hypotonus as well as problems related to movement fluency, axial control, balance, coordination, and speech (Saavedra et al., 2009; Singer et al., 2010; Sveljo et al., 2014). Individuals with DS also exhibit diffuse electrical functioning during a cognitive activity (Flórez et al., 1997; Santos et al., 2010) as well as difficulties selecting and directing a neurophysiological stimulus due to nerve connection fatigue (Luria and Tsvetkova, 1964; Bomono and Rosseti, 2010). Problems related to balance and agility due to diminished primitive reflexes and delays in motor and cognitive development constitute a barrier to the acquisition of fundamental skills (O'shea et al., 2014).

Transcranial direct current stimulation (tDCS) is a noninvasive brain stimulation technique with promising results with regards to motor learning when used in physical rehabilitation. Is capable of modulating the excitability of the central nervous system including neurons, making it a useful tool in the rehabilitation of patients with neurological disorders (Grecco et al., 2014a; Lopes et al., 2018a,b; Miziara et al., 2018; Duarte et al., 2019; Lazzari et al., 2019). The technique consists of the administration of a low-intensity, monophasic, electrical current through silicone-sponge surface electrodes moistened in saline solution and positioned over the scalp. The effects of tDCS are obtained by the movement of electrons due to differences in positive and negative charges. Numerous benefits are reported, but the main effects stem from the voltage-dependent inhibition or activation of N-methyl-D-aspartate (Nitsche and Paulus, 2001; Nitsche et al., 2004; Lefebvre et al., 2005; Wagner et al., 2007a; Kuo et al., 2008; Monte-Silva et al., 2009; Stagg et al., 2012; Chung and Warren, 2015; Miziara et al., 2018). According to the literature, tDCS has beneficial modulatory effects on cortical function (neuromodulation) (Nitsche et al., 2004; Lefebvre et al., 2005; Kuo et al., 2008; Monte-Silva et al., 2009; Chung and Warren, 2015), which promotes an increase in local synaptic efficacy and alters the maladaptive plasticity pattern that emerges after a cortical injury (Nitsche et al., 2004; Lefebvre et al., 2005; Wagner et al., 2007a; Kuo et al., 2008; Monte-Silva et al., 2009; Stagg et al., 2012; Chung and Warren, 2015). The results of clinical trials demonstrate the considerable potential of this treatment modality for individuals with neurological disorders (Nitsche and Paulus, 2001; Nitsche et al., 2004; Lefebvre et al., 2005; Wagner et al., 2007a; Kuo et al., 2008; Monte-Silva et al., 2009; Stagg et al., 2012; Chung and Warren, 2015; Miziara et al., 2018). This technique promotes a subtle change in cortical excitability, altering the potential of the cell membrane, either facilitating or hindering depolarization (Chung and Warren, 2015). Moreover, tDCS can be used concomitantly with physical therapy, which may enhance and prolong motor gains, optimizing the functional outcome due to the potentiation of neuroplastic changes (Nitsche and Paulus, 2001; Nitsche et al., 2004; Lefebvre et al., 2005; Wagner et al., 2007a; Kuo et al., 2008; Monte-Silva et al., 2009; Stagg et al., 2012; Chung and Warren, 2015; Miziara et al., 2018).

Xbox Kinect game activities are effective and lead to improvements in sensory-motor, adaptive, and functional aspects (Wagner et al., 2007a; Stagg et al., 2012). The results obtained with the use of Xbox Kinect games are believed to be related to training in an interactive environment with a broad gamut of activities and scenarios, multiple sensory channels, and the creation of exercises that could be beneficial to the rehabilitation process of patients with neurological disorders (Jung et al., 2009; Pavão et al., 2014).

The determination of the effects of tDCS combined with Xbox Kinect games, especially in terms of motor adaptation, requires the use of three-dimensional (3D) movement analysis (Sveistrup, 2004; Gamberini et al., 2006; Jung et al., 2009), which is a powerful tool for the quantitative analysis of movement and is considered the gold standard for the evaluation of the lower limbs during gait (Pavão et al., 2014). However, the analysis of the upper limbs is technically more challenging due to the non-cyclic movements and the complexity of shoulder motion (Gamberini et al., 2006). In addition to joint kinematics, spatiotemporal variables, such as the duration, velocity, smoothness, and trajectory of a motor task, furnish important quantitative information on the quality of upper limb movement (Sveistrup, 2004; Gamberini et al., 2006; Jung et al., 2009; Biddiss and Beng, 2010; Damian et al., 2014).

Therefore, this study aimed to determine whether tDCS can enhance the effects of motor upper limb training involving Xbox Kinect game activities in children with Down syndrome.

## Materials and Methods

### Case Report

The study received approval from the Human Research Ethics Committee of Nove de Julho University (São Paulo, Brazil) (certificate number: 1.540.113), was conducted in accordance with the ethical principles established in the 1964 Declaration of Helsinki, and was registered with the Brazilian Clinical Trials Registry (N° RBR3PHPXB). All legal guardians agreed to the participation of the child by signing a statement of informed consent prior to the onset of the study.

**Figure 1:**
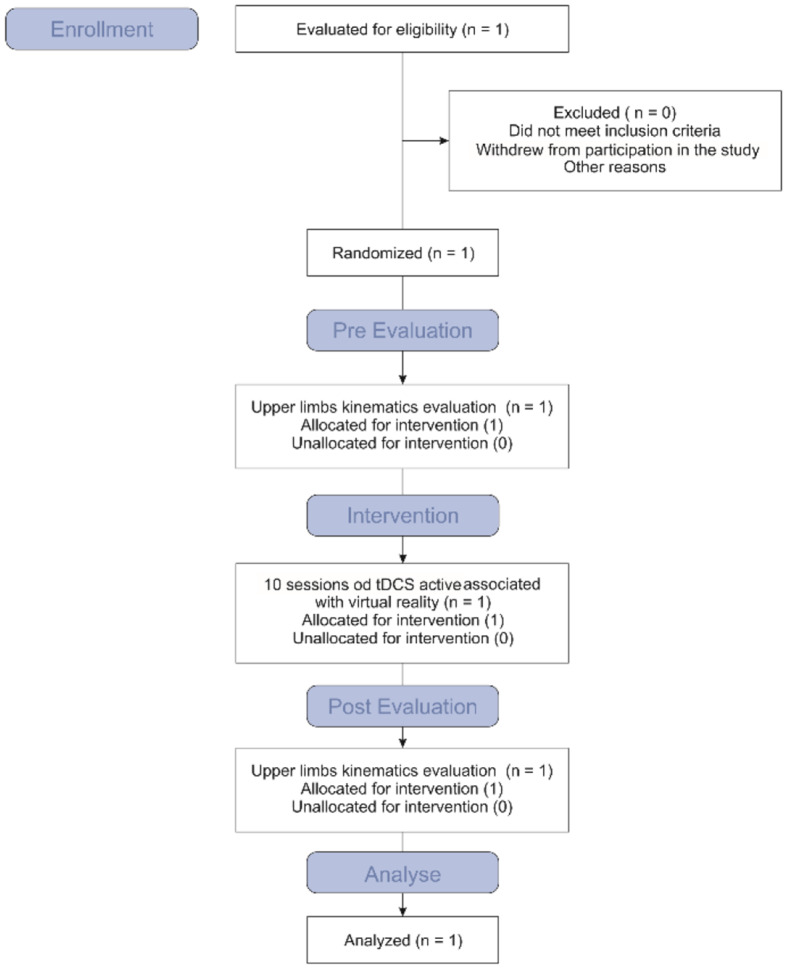
Study flowchart.

This study is a case report of the effects of tDCS combined with Xbox Kinect game training on kinematic variables during the execution of an upper limb motor task (Lebiedowska et al., 2004; Santos et al., 2015; Lopes et al., 2017a,b) (Figure 1). The eligibility criteria were as follows: (1) diagnosis of DS; (2) adequate understanding and cooperation during the procedures; (3) aged six to 12 years; (4) impairment of upper limb motor coordination; and (5) statement of informed consent signed by a legal guardian. The exclusion criteria were as follows: (1) history of surgical procedures in the 12 months prior to the onset of the training sessions, (2) orthopedic deformity of the lower limbs or spinal column with indication for surgery, (3) epilepsy, (4) metal implant in the skull or hearing aids, (5) associated neurological disorder, and (6) the use of a pacemaker (Santos et al., 2015).

An eight-year-old child underwent an evaluation using the Wechsler Intelligence Scale for Children—Third Edition (WISC-III) (Cruz, 2005), which yielded the following results: verbal communication: 24/100; performance: 34/100; total score: 58/100. The child had an intelligence quotient of 70, indicating a cognitive age of five years. According to the medical history, the mother's pregnancy progressed normally until 31 weeks when the child was born prematurely, weighing 1,800 g. The karyotype exam was performed to investigate and confirm the diagnosis of DS.

The child has received physical therapy since birth. During the first years of life, the child exhibited delayed motor development, hypotonia, and delayed ambulation. At the time of the study, the child exhibited compromised functional skills during the execution of two-handed tasks due to mental disability.

**Figure 2:**
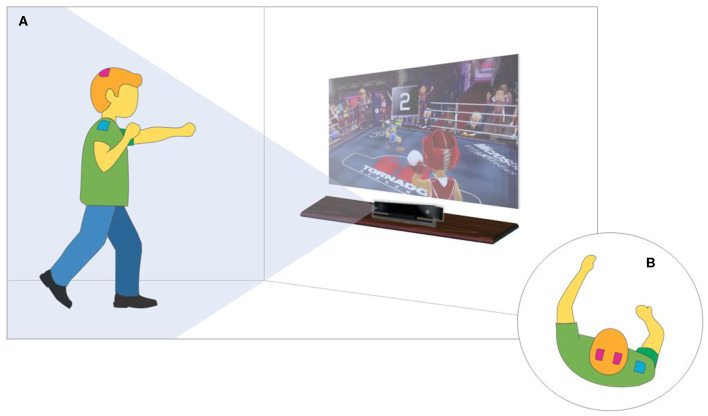
XBOX-KINECT game training and tDCS. **(A)** Virtual Environment Overview. **(B)** top view focusing on tDCS positioning.

The therapeutic intervention consisted of a protocol involving the combination of tDCS and the Xbox Kinect game (Figure 2) following safety procedures described in the literature for the use of tDCS on the pediatric population. The 20-min training sessions were held three times a week on non-consecutive days for a total of 10 sessions (Santos et al., 2015; Lopes et al., 2017a,b). Stimulation was administered using a tDCS device (DC-Stimulator; neuroConn GmbH, Ilmenau, Germany) with three sponge (non-metallic) electrodes measuring 5 × 5 cm (25 cm^2^) soaked in saline solution (Cruz, 2005; Nasseri et al., 2015; Santos et al., 2015; Grecco et al., 2017; Lopes et al., 2017a,b). The anodes were positioned over C3 and C4 (10–20 international electroencephalogram system), corresponding to the primary motor cortex (M1) (Cruz, 2005; Santos et al., 2015; Moura et al., 2016, 2017; Grecco et al., 2017; Lopes et al., 2017a,b). The cathode was positioned over the belly of the deltoid muscle.

The major limitation observed during the execution of the training protocol was the electrode montage for tDCS (Cruz, 2005; Nasseri et al., 2015; Santos et al., 2015; Grecco et al., 2017; Lopes et al., 2017a,b) as few studies have involved individuals with DS. We first performed a search of the literature and found that studies using kinematic variables for the evaluation and tDCS during the training protocol opted for C3 as the stimulation site for modulating the excitability of the motor region. To further add to the enhancement, we made the decision to stimulate two points of the motor cortex using the 10–20 electroencephalogram system, as suggested by Lopes et al. (2017a). Thus, the anodes were positioned over C3 and C4, and the cathode was positioned over the belly of the deltoid muscle (Lopes et al., 2017a). We then noticed positive points, such as an increase in the stimulation of the motor region, as well as a negative point, namely, a larger field of stimulation increased the odds that the individual would perceive the stimulation. Greater tactile sensation was perceived at different moments during the protocol in comparison to previous studies by our research group involving the use of only one anodic electrode over the scalp (Cruz, 2005; Nasseri et al., 2015; Santos et al., 2015; Grecco et al., 2017; Lopes et al., 2017a,b). Despite the tactile sensation, we had no problems regarding the execution of the protocol.

A current considered safe for the pediatric population (1 mA) (Santos et al., 2015; Moura et al., 2016) was administered for 20 consecutive minutes, during which the child underwent upper limb motor training with the aid of the Xbox 360™ (Microsoft, Redmond, WA, USA) and the Kinect™ motion detector (Microsoft). The activity was performed in a specific room (2.5 × 4.0 m) at the Integrated Human Movement Lab. To provide adequate visual and auditory stimuli, the game was shown on a projection screen measuring 200 × 150 cm, and stereo speakers were used (Figure 2).

The kinematic data were captured using the SMART-D 140® optoelectronic system (BTS Bioengineering, Italy) composed of eight cameras sensitive to infrared light, with a sampling frequency of 100 Hz and synchronized video system. As reported in the literature (Menegoni et al., 2009; Cimolin et al., 2012). Eighteen reflective markers were positioned on prominent bone points of the upper limb to enable the detection of the trajectory of the reaching movement.

**Figure 3:**
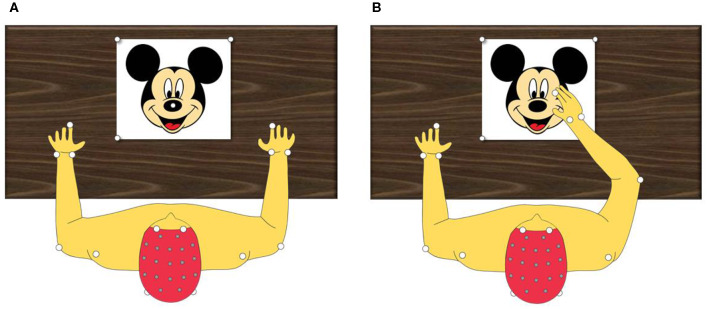
Kinematic evaluation protocol. **(A)** Top view of static evaluation. **(B)** Top view of evaluation during range movement.

Kinematic variables (joint angles, movement duration, and velocity) were evaluated during a reaching task. For the task, the child sat comfortably on an adjustable chair with the elbows flexed at 90° and the reaching hand resting on a mark positioned in front of the child on a table. A target was positioned at 80% of the arm length. The task involved touching the target with the tip of the index finger with precision at a comfortable velocity and returning the hand to the initial position (Figure 3).

This task was chosen because it was considered representative of everyday functional activities, with similar tasks having been described in previous studies examining upper limb movement in different pathologies (Chang et al., 2005; Mackey et al., 2005, 2006; Caimmi et al., 2008; Menegoni et al., 2009; Cimolin et al., 2012; Camerota et al., 2014).

Each session was composed of six reaching movements—three with the right limb and three with the left limb. The biomechanical model, filtering of the data. and processing of the variables were performed using the SMART Analyzer (BTS Bioengineering) (Menegoni et al., 2009; Cimolin et al., 2012; Camerota et al., 2014; Moura et al., 2017). Mean and standard deviation values were obtained for the following variables: movement duration (total time required to execute the entire reaching task), mean movement velocity (determined using the marker positioned on the index finger), range of motion (ROM) of the elbow and shoulder (calculated as the difference between the maximum and minimum angles of the elbow and shoulder in the sagittal and frontal planes), and smoothness and precision of the movement. The smoothness and precision of the movement were represented by the following indices: (1) index of curvature (IC), calculated as the ratio of the fingernail 3D-path length to the linear distance between the initial and final pointing position; it is representative of movement smoothness during the ongoing phase; (2) average jerk (AJ), derived from the derivative of the acceleration (i.e., jerk) of the marker on the fingernail; it has been shown that the AJ index decreases with increased smoothness of movement; it is often used as a measure of the quality of selective motor control;(Menegoni et al., 2009; Camerota et al., 2014); and (3) the number of movement units (NMU), computed as the number of velocity peaks that exceed the 10% of peak velocity; hence, NMU was aimed at capturing the number of online corrections performed by the subject during the ongoing phase (Chang et al., 2005; Mackey et al., 2005, 2006; Caimmi et al., 2008; Menegoni et al., 2009; Cimolin et al., 2012; Camerota et al., 2014).

Upper limb kinematics were evaluated during three sessions: pre-intervention (before the onset of the treatment protocol involving 10 sessions of tDCS combined with the Xbox Kinect game at a frequency of three times per week on non-consecutive days), post-intervention (immediately after the end of the 10-session protocol), and follow-up (one month after the end of the sessions; the individual did not receive any type of treatment in this period).

The mean values for all the previously defined variables were computed from the three repetitions of each trial for each limb. The results were then compared to the values of the age-matched control group (CG). The significant level was set at the alpha value of 5%.

## Results and Discussion

tDCS currently occupies an important place in studies addressing neuromotor rehabilitation due to its potential to optimize the results of therapy (Cruz, 2005; Lopes et al., 2017a,b). When used properly, this technique induces changes in neural excitability and affects local plasticity (Lopes et al., 2017b). However, proper use depends on the location and polarity of the electrodes as well as the intensity of the current administered to the cortex (O'shea et al., 2014).

The literature reports the use of diverse interventions to enhance upper limb motor learning and favor manual abilities (Monteiro et al., 2017; Moura et al., 2017). One such intervention is the use of interactive games in computer-simulated environments, which favors motor learning, and assists in the training of cognitive skills. When selected based on executive properties, Xbox Kinect activities favor a gamut of objectives related to motor and cognitive learning (Gamberini et al., 2006; Jung et al., 2009; Biddiss and Beng, 2010; Damian et al., 2014). The combination of anodal tDCS administered over the primary motor cortex during motor training involving Xbox Kinect games has been used in several studies with the aim of improving gait and upper limb movements (Grecco et al., 2014b, 2017; Moura et al., 2016; Duarte et al., 2019; Lazzari et al., 2019). This stimulation method combined with the executive potential of the game can modulate cortical excitability, thereby enhancing the effects obtained with training. Moreover, the maintenance of these results is also promising, as improvements in functional performance and kinematic variables are maintained for at least one month after the rehabilitation protocol, as demonstrated in the present case report.

Limited arm use and impaired motor coordination exert a negative impact on activities of daily living as well as functioning in general. This is a significant factor in DS, as a limited arm movement leads to difficulties in activities involving reaching, grasping, and handling objects. Such problems compromise one's functional performance, with negative impacts on independence, mobility, and self-care (Nasseri et al., 2015; Moura et al., 2016; Grecco et al., 2017). Rehabilitation involving neuromodulation tends to increase local synaptic efficacy, altering the plasticity pattern in the cortex and enhancing the performance of a motor task. Such stimulation enables a change in the dysfunctional pattern of excitability through the activation of specific neural networks, favoring neuromodulation (Nitsche and Paulus, 2001; Wagner et al., 2007b). Studies involving neuromodulation over the primary motor cortex in stroke survivors report improvements in kinematic variables of upper limb function (active movements of the wrist and fingers), movement velocity, active movements of the ankle, and general motor function (Madhavan et al., 2011; Gillick et al., 2015; Moura et al., 2017). However, there are no previous reports of this technique in individuals with DS.

The aim of the present study was to induce changes in motor cortex excitability using a tDCS protocol combined with motor training induced/motivated by an Xbox Kinect game. With the proposed protocol, tDCS is believed to optimize motor training, leading to modifications in kinematic variables during the execution of a motor task.

Regarding upper limb kinematics (Table 1), the patient was slower in performing the movement with the right arm during the pre-intervention evaluation, particularly during the going and adjusting phases (Going MD, Adjusting MD, Total MD, and MV indices). Smoothness of movement was characterized by a higher IC and greater number of movement units (NMU index) compared to the reference CG (Menegoni et al., 2009; Cimolin et al., 2012). In the ROM analysis, the shoulder and elbow exhibited high excursion in the sagittal plane. The strategy used for the non-dominant limb was closer to that chosen by the CG (Menegoni et al., 2009; Cimolin et al., 2012). The only differences were the duration of the adjusting phase (Adjusting MD index) and the IC, which were higher than that of the controls. In contrast, the ROM for shoulder abduction-adduction was lower compared to that of the CG (Menegoni et al., 2009; Cimolin et al., 2012).

**Table 1:** Kinematic measures (mean and standard deviation) for child with Down syndrome at three evaluation times (pre-intervention, post-intervention, and follow-up) and compared to control group.

	***Pre-intervention***		***Post-intervention***		***Follow-up***		***CG^**40**^***
	**Dominant side**	**Non-dominant side**	**Dominant side**	**Non-dominant side**	**Dominant side**	**Non-dominant side**	
**Movement duration (MD) (s)**							
Total MD	3.05 (0.19)	2.05 (0.11)	1.58 (0.08)	1.46 (0.08)	1.52 (0.05)	1.24	1.97 (0.15)
Going MD	1.14 (0.22)	0.71 (0.09)	0.60 (0.07)	0.54 (0.03)	0.44 (0.03)	0.38 (0.03)	0.82 (0.17)
Adjusting MD	1.11 (0.19)	0.73 (0.14)	0.42 (0.08)	0.44 (0.12)	0.55 (0.08)	0.45 (0.16)	0.28 (0.15)
Returning MD	0.80 (0.17)	0.62 (0.07)	0.56 (0.08)	0.48 (0.04)	0.52 (0.06)	0.41 (0.06)	0.75 (0.12)
**Movement smoothness and precision**							
IC	1.40 (0.19)	1.29 (0.22)	1.17 (0.07)	1.07 (0.05)	1.10 (0.06)	0.71 (0.09)	1.09 (0.15)
AJ (mm/s^3^)	236.22 (13.09)	257.48 (32.56)	217.39 (10.52)	209.05 (15.54)	275.86 (67.71)	225.18 (9.07)	229.62 (14.60)
NMU	14 (1.54)	5.6 (1.19)	3.4 (0.98)	3.2 (0.93)	2.0 (0.48)	2.0 (0.42)	2.77 (1.45)
**Velocity (m/s)**							
MV	0.37 (0.06)	0.53 (0.12)	0.59 (0.13)	0.54 (0.07)	0.93 (0.04)	0.85 (0.06)	0.53 (0.07)
**Angles (°)**							
ROM shoulder flex-extension	50.4 (11.20)	22.1 (9.71)	54.3 (11.10)	57.6 (18.4)	38.5 (4.8)	50.1 (9.6)	27.26 (9.90)
ROM shoulder ab-adduction	17.3 (6.03)	8.4 (3.82)	14.7 (7.50)	26.6 (9.2)	15.7 (5.2)	25.3 (2.2)	21.5 (5.90)
ROM elbow flex-extension	26.8 (12.20)	15.7 (6.60)	14.4 (5.31)	22.6 (7.5)	14.5 (4.5)	14.3 (3.3)	15.32 (3.51)

*MD, Movement Duration; MMV, Mean Movement Duration; IC, Index of Curvature; AJ, Average Jerk; NMU, Number of Movement Units; MV, Mean Velocity; ROM, Range of Motion. CG, Control Group*.

Previous studies report motor limitations in children with DS during upper limb activities (Petuskey et al., 2007,?; Williams et al., 2010; Johnston et al., 2011; Cao et al., 2015). In one study proposing a protocol to quantify functional motor limitation in individuals with DS during the execution of a set of ROM tasks, the authors suggested that the increased time required to perform the tasks in the DS group is associated with low muscle tone and limited motor coordination, which are common among individuals with DS (Volman et al., 2007). According to the authors, this result is also directly associated with the reduced movement velocity commonly found as a compensatory pattern in DS. Moreover, even in cases for which the clinical evaluation is good, general joint stiffness during arm abduction contributes to poor coordination in individuals with DS, diminishing maximum and minimum joint angles (Valvano et al., 2017).

Analyzing kinematic variables in individuals with DS during two upper limb functional tasks, the authors of another study attributed the significant limitations to deficits in motor planning and movement execution. According to the authors, characteristics such as decreased manual dexterity, the significantly longer time required to complete the task after contact with the object and the delayed onset of late movement are indicative of poorer motor planning in children with DS compared to those with typical development (Hartman et al., 2010).

In the post-intervention evaluation, the dominant limb showed improvements in the duration of all phases, total movement duration, and velocity. Moreover, reductions were found in the IC, AJ, and NMU indices, indicating a smoother, less segmented trajectory. The ROM of the elbow also improved. For the left arm, reductions occurred in the duration of all phases as well as the IC and AJ indices. Shoulder ROM (flexion-extension and abduction-adduction) also increased (Rab et al., 2002; Horvat et al., 2013).

At the one-month follow-up, further improvements were evident on the dominant side in terms of Going MD and MV; the shoulder flexion-extension ROM was reduced, reaching a value closer to that found in the CG (Menegoni et al., 2009). For the non-dominant limb, improvements were found in the IC and MV indices and a reduction was found in the elbow flexion-extension ROM, indicating improvement (Rab et al., 2002; Menegoni et al., 2009; Cimolin et al., 2012; Horvat et al., 2013; Camerota et al., 2014; Moura et al., 2017).

Reductions in the total duration of a reaching movement after tDCS were also observed in children with hemiparetic cerebral palsy in the study by Moura et al. (2017). We believe that the increased shoulder ROM, reduced elbow ROM, reduced movement duration, and increased movement speed could signify a reduction in joint stiffness. Moreover, less segmented trajectories are characteristic of better movement organization, i.e., better motor planning (Galli et al., 2008; Jover et al., 2010; Grecco et al., 2014a; Moura et al., 2017).

Based on previous studies and the present results, we suggest that the 10-session tDCS protocol using the Xbox Kinect game is a valuable therapeutic option for the rehabilitation of upper limb function. However, studies have shown increased benefits when tDCS combined with motor training is used in a larger number of sessions (Dehema et al., 2018). Moreover, the long-term retainment of improvements demonstrated here and in previous studies (Braendvik et al., 2010; Karok et al., 2017) lends further support to tDCS as a promising tool for neurorehabilitation.

Further investigations with larger samples are needed for an effective assessment of this intervention. It would also be interesting to assess whether the improvements are maintained over time without further changes. Nevertheless, the fact that improvements occurred in upper limb kinematics after the treatment period suggests that anodal tDCS combined with upper limb motor training using Xbox Kinect activities is a promising intervention for improving upper limb function in patients with DS.

## Conclusion

The present case report offers preliminary data from a protocol study, and the results seem to confirm the notion that anodal tDCS combined with upper limb motor training leads to improvements in different kinematic variables.

## Ethics Statement

The studies involving human participants were reviewed and approved by Ethics committee (certificate number: 1.540.113). Written informed consent to participate in this study was provided by the participants' legal guardian/next of kin.

## Author Contributions

JL and IM: organization and execution of research project, data collection and interpretation, statistical analysis, writing, critical review, and revision of the manuscript. VC: assistance in the interpretation of the data and statistical analysis. VC, MG, and CO: final correction.

## Conflict of Interest

The authors declare that the research was conducted in the absence of any commercial or financial relationships that could be construed as a potential conflict of interest.

## Abbreviations

DS: Down Syndrome
TDCS: Transcranial Direct Current Stimulation
WISC-III: Wechsler Intelligence Scale for Children – Third Edition
CG: Control Group
MD: Movement Duration
MMV: Mean Movement Duration
IC: Index of Curvature
AJ: Average Jerk
NMU: Number of Movement Units
MV: Mean Velocity
ROM: Range of Motion
CNPq: Conselho Nacional de Desenvolvimento Científico e Tecnológico
CAPES: Coordenação de Aperfeiçoamento de Pessoal de Nível Superior.
